# PAR1 signaling on tumor cells limits tumor growth by maintaining a mesenchymal phenotype in pancreatic cancer

**DOI:** 10.18632/oncotarget.25880

**Published:** 2018-08-10

**Authors:** Cansu Tekin, Kun Shi, Joost B. Daalhuisen, Marieke S. ten Brink, Maarten F. Bijlsma, C. Arnold Spek

**Affiliations:** ^1^ Amsterdam UMC, University of Amsterdam, Center of Experimental and Molecular Medicine, Amsterdam, The Netherlands; ^2^ Amsterdam UMC, University of Amsterdam, Laboratory for Experimental Oncology and Radiobiology, Cancer Center Amsterdam, Amsterdam, The Netherlands; ^3^ Oncode Institute, Amsterdam, The Netherlands

**Keywords:** pancreatic cancer, PAR1, thrombin receptor, EMT

## Abstract

Protease activated receptor-1 (PAR1) expression is associated with disease progression and overall survival in a variety of cancers. However, the importance of tumor cell PAR1 in pancreatic ductal adenocarcinomas (PDAC) remains unexplored. Utilizing orthotopic models with wild type and PAR1-targeted PDAC cells, we show that tumor cell PAR1 negatively affects PDAC growth, yet promotes metastasis. Mechanistically, we show that tumor cell-specific PAR1 expression correlates with mesenchymal signatures in PDAC and that PAR1 is linked to the maintenance of a partial mesenchymal cell state. Indeed, loss of PAR1 expression results in well-differentiated pancreatic tumors *in vivo*, with enhanced epithelial characteristics both *in vitro* and *in vivo*. Taken together, we have identified a novel growth inhibitory role of PAR1 in PDAC, which is linked to the induction, and maintenance of a mesenchymal-like phenotype. The recognition that PAR1 actively limits pancreatic cancer cell growth suggest that the contributions of PAR1 to tumor growth differ between cancers of epithelial origin and that its targeting should be applied with care.

## INTRODUCTION

Pancreatic ductal adenocarcinoma (PDAC) is a highly aggressive disease with an extremely low survival rate (5-year survival ~7.7%) [1, 2]. This high mortality rate is largely due to late diagnosis with the vast majority of patients presenting with locally advanced or metastatic disease, and only around 20% of the patients are eligible for surgical resection. Progress in improving survival has been slow, and current treatment options are severely inadequate. The only noteworthy progress has been in lowering mortality rates for patients undergoing resections, and a small prolongation and improved quality of life in patients with unresectable disease by chemotherapeutic agents [3]. Novel combination therapies, like for instance FOLFIRINOX [4] or gemcitabine with Nab-paclitaxel [5], are superior over single-drug regimens but even in the specific group of patients eligible for treatment the survival benefit is limited.

A key factor responsible for the poor prognosis in PDAC is a high propensity for epithelial-to-mesenchymal transition (EMT) of pancreatic cancer cells [6]. EMT, a biological process where epithelial cells morphologically and phenotypically transition into mesenchymal cells [7], is associated with invasion and metastasis in various cancers [8–10]. Loss of epithelial characteristics, as revealed by a loss of E-cadherin expression in a Snail and/or zinc finger E-box-binding homeobox 1 (ZEB1) dependent manner [11, 12], correlates with poor prognosis and poor therapeutic outcome [13, 14]. Importantly, suppression of EMT enhances therapeutic efficacy and survival in a murine pancreatic cancer model [15].

Protease activated receptor 1 (PAR1), also known as the thrombin (F2) receptor, is a seven-transmembrane G-coupled receptor. As implied by its name, PAR1 is activated by proteolytic cleavage of a N-terminal extracellular region by proteases such as thrombin, activated protein C and matrix metalloproteases [16]. Interestingly, PAR1 expression is increased in breast, lung, ovarian, and prostate cancer [17–20] and PAR1 expression correlates with poor prognosis in breast [21] and lung cancer [22]. In line with these clinical data pointing to a tumor-promoting effect of PAR-1, experimental studies underscore the tumor-promoting actions of activated PAR1. For instance, PAR1 expression is shown to be required and sufficient for tumor growth in a breast carcinoma xenograft model [17]. Moreover, pharmacological PAR1 inhibition inhibited lung tumor growth in nude mice [18]; PAR1 silencing decreased tumor growth and metastasis to the lung in a murine melanoma model [23]; and PAR1 inhibition in giant cell tumor of bone restrained tumor growth *in vivo* [24]. In the setting of pancreatic cancer, we recently showed that genetic ablation of PAR1 in the pancreatic stroma impeded tumor growth and metastasis [25] suggesting that PAR1 expression contributes to poor prognosis in pancreatic cancer.

In this manuscript, we addressed the hypothesis that PAR1 could be a prognostic marker for PDAC. However, we find that the survival of PDAC patients is not associated with PAR1 expression in bulk tumor tissue. We explain this by the observation that tumor cell-specific PAR1 expression is linked to the maintenance of a mesenchymal-like cell state. In an orthotopic pancreatic cancer model, the loss of tumor cell PAR1 induces well-differentiated tumors with increased epithelial characteristics, and enhanced tumor growth. We thus conclude that tumor cell PAR1 actively limits the growth of PDAC likely by playing a role in the induction and maintenance of a partial mesenchymal phenotype in PDAC.

## RESULTS

### Bulk tumor PAR1 expression does not associate with prognosis in PDAC

Previous work on PAR1 has demonstrated a role for PAR1 in tumor progression in different tumor types leading to poor prognosis in patients with high PAR1 expression levels [17, 21, 22, 25, 26]. Therefore, we hypothesized that PAR1 expression also holds prognostic value in PDAC. To assess this hypothesis, Kaplan–Meier survival analysis was performed on four PDAC gene expression sets dichotomized by median PAR1 expression. Surprisingly, PAR1 expression did not associate with overall survival in any of the expression sets (Supplementary Figure 1A–1D). However, given that PAR1 expression in these sets is the cumulative expression obtained from tumor cells, stromal content, and possibly adjacent non-tumor tissue, we reasoned that further analyses should address if PAR1 signaling in tumor and stromal compartments contribute differently to tumor growth.

### PAR1 regulates tumor cell differentiation and proliferation

**Figure 1:**
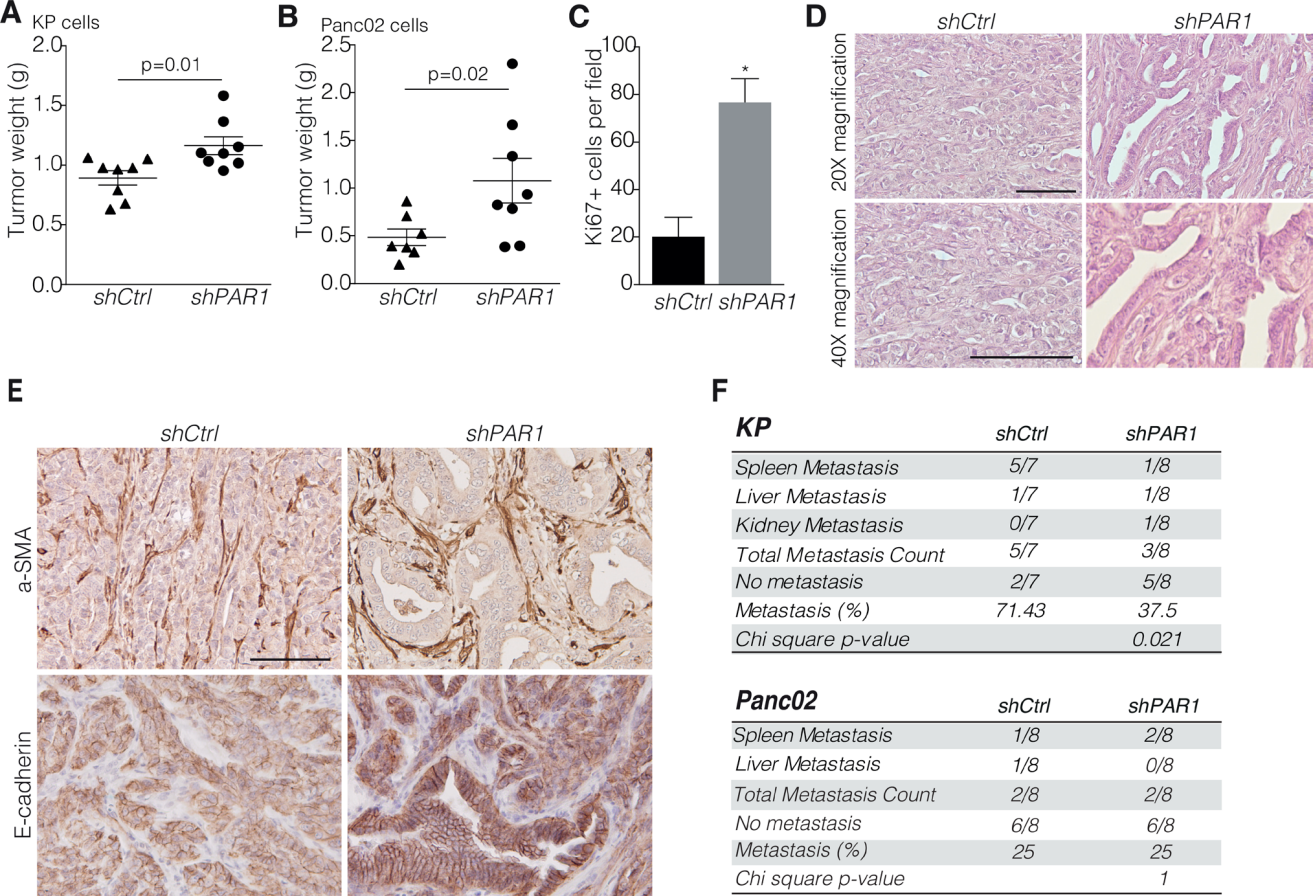
PAR1 negatively regulates tumor differentiation and growth Orthotopic inoculation of (**A**) shCtrl (*n* = 8) and shPAR1 (*n* = 8) KP cells and (**B**) shCtrl (*n* = 7) and shPAR1 (*n* = 8) Panc02 cells. Symbols show individual samples. Error bars show mean ± SEM: Mann-Whitney test (two-tailed). (**C**) For the KP model, Ki67+ counts per field (at 200X magnification) for shCtrl (*n* = 5) and shPAR1 (*n* = 5) tumors, error bars show mean ± SEM. Mann-Whitney (two-tailed), ^****^< 0.0001. (**D**) KP shCtrl (left) and shPAR1 (right) tumor staining with hematoxylin and eosin at 200× (upper panels) and 400× (lower panels) magnification. (**E**) KP shCtrl (left) and shPAR1 (right) tumor immunohistochemistry with a-SMA (upper panels) and E-cadherin (lower panels) staining. Scale bar is 200 μm. (**F**) Macro-metastasis scores of the KP and Panc02 models, for shCtrl and shPAR1 animals. Group differences were tested with chi-square distribution tests (for KP group *p* = 0.021, for Panc02 group *p* = 1).

Previously, we showed that PAR1 expression in PDAC stroma drives tumor progression [25] and the lack of association between PAR1 and overall survival in PDAC patients lead us to reason that tumor cell-specific PAR1 might counteract the tumorigenic stromal PAR1 activity and reduces the detrimental effect on overall survival. To assess the effect of PAR1 expression on tumor cells and the suspected counterbalancing activity, cells derived from p48-CRE/LSL-KRAS/P53^flox/flox^ mice (named KP hereafter) and Panc02 murine pancreatic cancer cells were transduced with short hairpin RNA against PAR1 (shPAR1) or with control short hairpin RNA (shCtrl). PAR1 knockdown was confirmed by measuring PAR1-dependent calcium fluxes as described before [27] (Supplementary Figure 2A and 2B). Importantly, PAR1 knockdown did not affect *in vitro* proliferation of both cell lines (Supplementary Figure 3A and 3B). After subsequent orthotopic engraftment to wildtype C57Bl/6 animals, shPAR1 knockdown cells formed significantly bigger tumors as compared to vector control cells (Figure 1A and 1B). Subsequent stainings for the proliferation marker Ki67 showed a higher density of Ki67 positive cells in shPAR1 tumors than in shCtrl tumors (Figure 1C). Histopathological examination of KP pancreatic cancer sections showed abundant ductal structures throughout the tumor in the shPAR1 group, whereas poorly differentiated tumors lacking apparent ductal structures were observed in the control group (Figure 1D). We next analyzed alpha smooth muscle actin (a-SMA); a marker for activated stromal fibroblasts, but did not find any difference in expression of this marker between shPAR1 and shCtrl tumors (Figure 1E), indicating that PAR1 knockdown on tumor cells does not effect stromal recruitment and activation. In contrast, expression and membrane localization of the epithelial marker E-cadherin was markedly increased in shPAR1 tumors as compared to shCtrl tumors (Figure 1E). Furthermore, in the shPAR1 KP engrafted animals significantly less macro-metastasis were found compared to shCtrl animals (Figure 1F), mainly to the spleen. Overall, these data thus suggest that tumor cell PAR1 contributes to enhanced mesenchymal features.

### PAR1 associates with tumor cell-intrinsic mesenchymal programs

**Figure 2:**
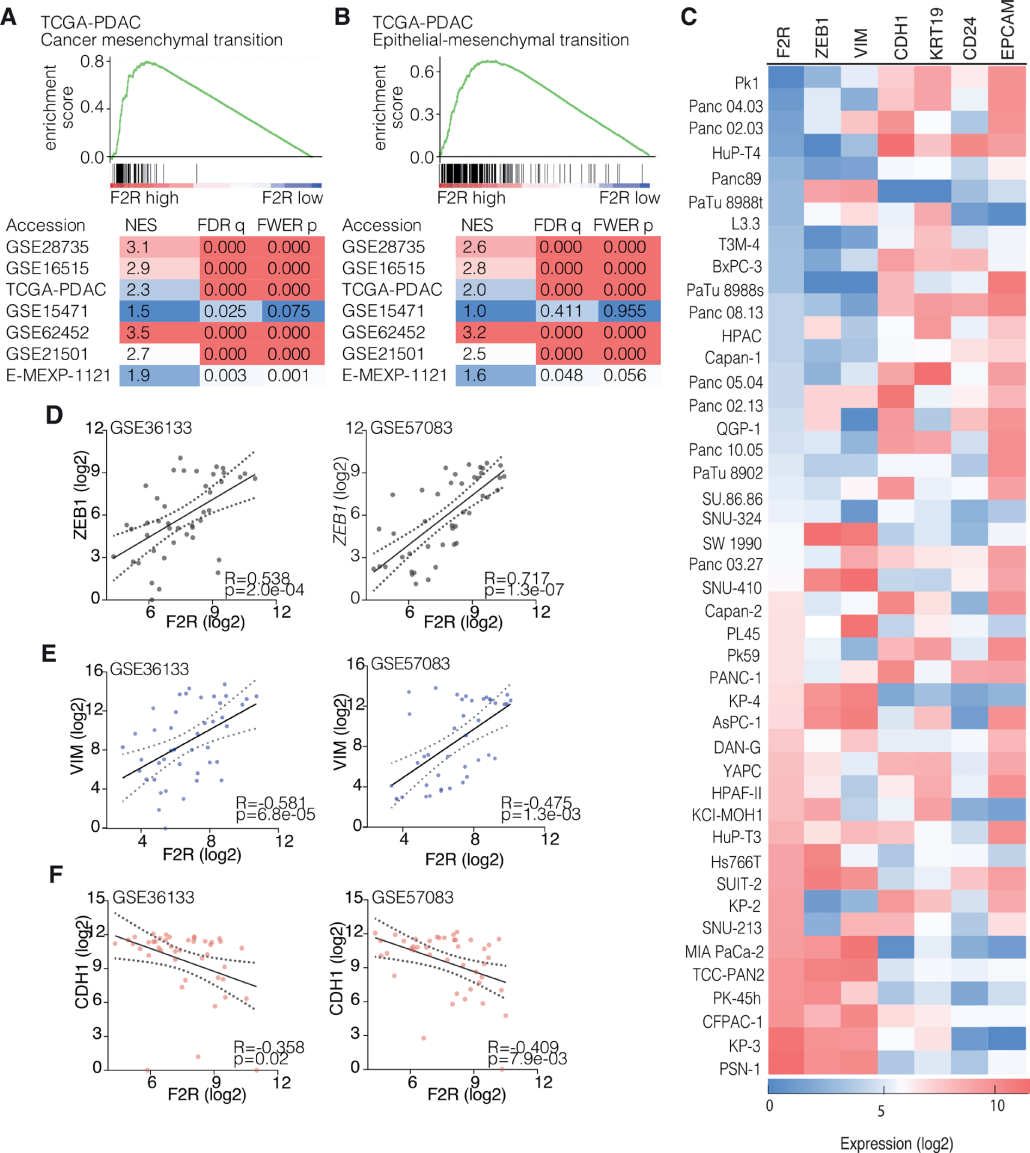
PAR1 expression correlates with EMT signatures (**A**–**B**) Gene Set Enrichment Analysis (GSEA) results of different PDAC gene expression sets with the EMT signature set (A) [35] and the EMT hallmark gene set (B) (Broad Institute). Enrichment plots are shown for both signature sets in the TCGA-PDAC expression dataset. Normalized Enrichment Score (NES), False Discover Rate (FDR) *q*-value and Family-Wise Error Rate (FWER) *p*-value are shown for each tested gene expression set. (**C**) Gene expression heat map of pancreatic cancer cell line expression from GSE36133 and GSE57083 for *F2R* (PAR1), *ZEB1*, *VIM*, *CDH1*, *KRT19*, *CD24* and *EPCAM*. Color coding of the heatmap is by log2 transformed gene expression. (**D**–**F**) Correlation of PAR1 expression in PDAC cell lines with ZEB1, Vim and CDH1 in the GSE36133 and GSE57083 datasets. Dots show expression levels for individual cell lines. 95% confidence interval and linear regression line are shown; p value is corrected for multiple testing (FDR correction).

To elucidate the mechanism through which PAR1 impacts on tumor cell differentiation, we performed gene set enrichment analysis (GSEA) [28] for mesenchymal cell state, and differentiation-related genes on PDAC gene expression sets. The analyses shows that high PAR1 expression was associated with a mesenchymal cancer signature, as well as with a hallmark epithelial-to-mesenchymal transition signature in all expression sets analyzed, including a micro-dissected tumor cell set. This suggests that tumor cell PAR1 expression is linked to a mesenchymal cell state in PDAC (Figure 2A and 2B). To further confirm that PAR1 activity on tumor cells is associated with a mesenchymal phenotype and with decreased epithelial characteristics, we correlated PAR1 expression with different epithelial and mesenchymal markers in a large panel of PDAC cell lines available in the GSE36133 and GSE57083 datasets. As mesenchymal markers, Zinc Finger E-Box Binding Homeobox 1 (*ZEB1)* and Vimentin *(VIM)* were used and E-cadherin (*CDH1*), cytokeratin 19 (*KRT19), CD24,* and Epithelial cell adhesion molecule (*EPCAM)* were used as epithelial markers. PAR1/*F2R* followed similar expression patterns with *ZEB1* and *VIM*, whereas PAR1 expression was inversely correlated with the epithelial markers (especially prominent for *CDH1, KRT19 and EPCAM*, see Figure 2C). Furthermore, quantitative correlation analysis confirmed the strong positive association of *F2R* with *ZEB1* (Figure 2D) and *VIM* (Figure 2E) and showed a negative correlation between *F2R* and *CDH1* (Figure 2F).

**Figure 3:**
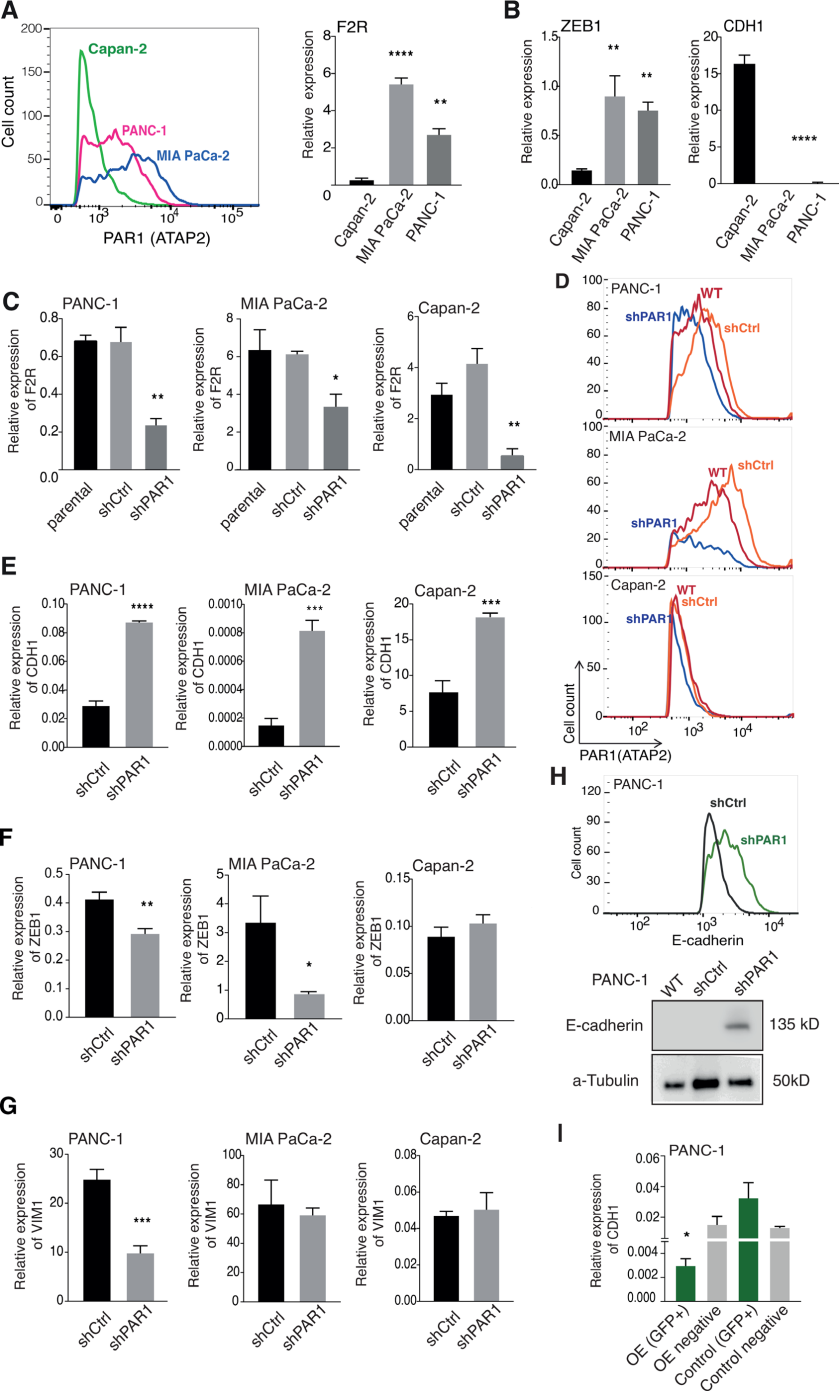
Short hairpin RNA mediated PAR1 knockdown induces E-cadherin and reduces ZEB-1 expression (**A**) PAR1 expression on MIA PaCa-2, PANC-1 and Capan-2 cell lines by flow cytometry and qPCR analysis. Flow cytometry histograms show cell counts versus PAR1 (ATAP-2)/APC intensity of the cell lines. Error bars in the qPCR graph show mean ± SEM: one-way ANOVA, ^****^<0.0001 (**B**) Relative mRNA expression levels for CDH1 and ZEB1 in three pancreatic cell lines (Capan-2, PANC-1, MIA PaCa-2). Symbols show triplicates. Error bars show mean ± SEM: one-way ANOVA, ^****^< 0.0001. (**C**) Relative PAR1 expression of shPAR1 and shCtrl transduced MIA PaCa-2, PANC-1 and Capan-2 cell lines in comparison to non-transduced (parent) cells. (**D**) Flow cytometry histogram showing the cell count versus PAR1 (ATAP-2)/APC intensity of wildtype (WT), shCtrl and shPAR1 cell lines. Initial gating was based on FCS and SSC for the main cell population and later FCS-H vs FCS-W for single cells. APC (PAR1) positive populations were gated on single cell population based on secondary antibody control. (**E**–**G**) Relative expression of CDH1 (E), ZEB1 (F), and VIM1 (G) in MIA PaCa-2, PANC-1 and Capan-2 shCtrl and PANC-1 shPAR1 cells. Symbols show quadruplicates. Error bars show mean ± SEM: Student's *t*-test, ^****^<0.0001. (**H**) Upper panel; flow cytometry histogram showing cell counts versus E-cadherin/Alexa 488 intensity of PANC-1 shCtrl (dark gray) and PANC-1 shPAR1 (green) cells. FITC (Alexa 488/E-cadherin) positive populations were gated on single cell population based on secondary antibody control. Lower panel; Western blot analysis for E-cadherin in PANC-1 wildtype, shCtrl and shPAR1 cells. a-Tubulin was used as loading control. (**I**) Relative E-cadherin mRNA expression levels in PANC-1 PAR1 OE (GFP+), OE negative (GFP negative) with Control (GFP+) and Control negative (GFP negative) cells. Symbols show quadruplicates. Error bars show mean ± SEM: one-way ANOVA, ^****^< 0.0001.

For conclusive evaluation of the *in silico* analysis, we next assessed PAR1 expression in pancreatic cancer cells isolated from tumors with different differentiation status [29] by flow cytometry and qPCR. These data confirmed that PAR1 levels were high in poorly differentiated MIA PaCa-2 cells; intermediate in moderately differentiated PANC-1 cells and relatively low in well-differentiated Capan-2 cells (Figure 3A). Subsequently, we performed qPCR-based transcript analysis for *ZEB1* and *CDH1* expression and, in line with the *in silico* data, ZEB1 expression patterns mirrored that of PAR1; high in MIA PaCa-2 cells and low in Capan-2 cells, whereas E-cadherin (CDH1) expression patterns were opposite to that of PAR1 (Figure 3B). To functionally ascertain that PAR1 activity is linked to ZEB1 upregulation and E-cadherin downregulation, we generated PAR1 shRNA knockdown MIA PaCa-2, PANC-1 and Capan-2 cell lines (Figure 3C, 3D). In agreement with the results above, PAR1 knockdown resulted in a significant increase in *CDH1* expression (Figure 3E) in all of the shPAR1cell lines compared to their controls. *ZEB1* expression was decreased in PANC-1 and MIA PaCa-2 shPAR1 cell lines compared to control cell lines and remained invariably low in the Capan-2 cell line (Figure 3F). Expression levels of the mesenchymal marker *VIM* were significantly decreased in PANC-1 shRNA cells but remained unchanged in high Vimentin expressing MIA PaCa-2 or low expressing Capan-2 cells (Figure 3G).

To confirm the expression data on the protein level, we analyzed E-cadherin levels on PANC-1 shCtrl and shPAR1 cells with flow cytometry, western blot, and immunofluorescence. We opted for PANC-1 cells in these experiments as they express intermediate levels of PAR1, E-cadherin and ZEB1 allowing efficient visualization of PAR1 knockdown, without confounding high endogenous ZEB1 or low/undetectably E-cadherin expression. Consistent with aforementioned results, all assays showed that PANC-1 shPAR1 cells had a markedly enhanced E-cadherin expression (Figure 3H and Supplementary Figure 4A). Increased E-cadherin expression was accompanied by decreased ZEB1 nuclear localization (Supplementary Figure 4A). Increased E-cadherin expression upon PAR1 knockdown in these cell lines led us question whether we can achieve the same affect with PAR1 inhibition. To test this, we treated PANC-1, MIA PaCa2 and Capan-2 cells with the PAR1 inhibitor Vorapaxar and determined E-cadherin surface expression by flow cytometry. In all cell lines analyzed, treatment with Vorapaxar increased E-cadherin expression (Supplementary Figure 4B).

Finally, we generated PAR1 overexpressing (PAR1-OE) PANC-1 cells to assess whether E-cadherin expression could be reduced. To this end, PANC-1 cells were transfected with PAR1-GFP or control-GFP plasmids after which cells were sorted based on GFP positivity (Supplementary Figure 5A, 5B). As expected, E-cadherin expression was nearly absent in GFP-positive PAR1-OE cells but not in GFP-positive control vector transfected cells, or in cells from the GFP-negative gates (Figure 3I). Taken together, we conclude that PAR1 levels are associated with a mesenchymal cell state and that loss of PAR1 enhances epithelial characteristics of pancreatic cancer cells, whereas gain of PAR1 diminishes such epithelial characteristics.

### PAR1 signaling drives tumor cell migration

**Figure 4:**
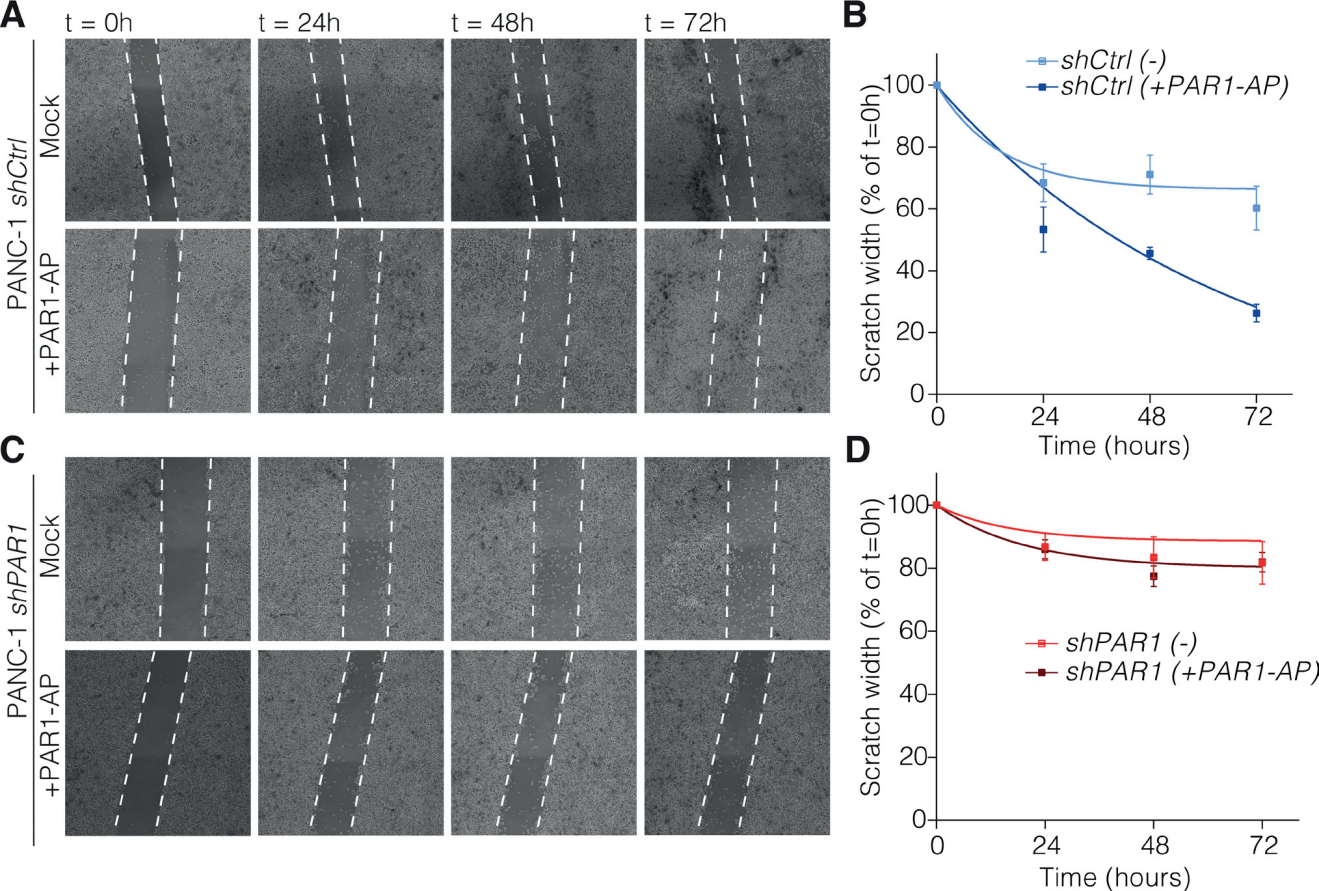
PAR-1 signaling contributes to tumor cell migration Scratch-healing assays were performed on PANC-1 shCtrl (**A**) and PANC-1 shPAR1 (**C**) with mock or 25 μM TFLLR-NH2 (PAR1 agonist peptide). At 80% confluency, cells were scratched on the vertical axis of the well with a sterile p200 tip. Images were taken at along the scratch every 24 hours up until 72 hours at 4× magnification on an EVOS FL Cell Imaging System. Scratch size was measured with ImageJ and calculated based on scratch size at t = 0 as 100% (*n* = 3). Scratch size over time for PANC-1 shCtrl (**B**) and PANC-1 shPAR1 (**D**) with or without the agonist peptide was calculated and put on non-linear one-phase decay curve in GraphPad Prism 7.0.

One of the functional outcomes of the transition to a more mesenchymal state is an enhanced migratory behavior and previously ZEB1 was reported to induce tumor cell invasion and enhanced metastatic potential [7, 30]. The above mentioned ZEB1 downregulation following PAR1 knockdown (shPAR1) in PANC-1 cells thus raises the question whether this affects the migratory capacities of the cells. To test this, we performed scratch/wound-healing assays with PANC-1 shCtrl and shPAR1 cells in the absence or presence of PAR1 agonist peptide TFLLR-NH2 (PAR1-AP) (Figure 4A and 4B). After 72 hours, PANC-1 shCtrl cells stimulated with PAR1-AP had higher migration rates than mock controls (Figure 4C), whereas mock or PAR1-AP treated PANC-1 shPAR1 cells had lower migration rates than shCtrl cells in all cases (Figure 4D). These findings show that activation of PAR1 induces the migration of PANC-1 cells. Although not as strong as agonist peptide induced shCtrl cells, mock treated shCtrl cells also present higher migration rates than shPAR1 cells both with or without PAR1-AP stimulation, meaning that endogenous PAR1 activity already operates the migratory activity of pancreatic cancer cells. Overall, our findings suggest that PAR1 activity on tumor cells promotes migration and exhibits enhanced metastatic potential.

## DISCUSSION

PAR1 is generally accepted to promote tumor progression [17, 25, 31], cancer cell invasion and metastasis [32, 33]. This notion is based on the fact that PAR1 expression is increased in various cancers types [17–20] and that PAR1 expression correlates with poor prognosis in breast [21] and lung cancer [22]. Experimental animal studies also support the notion of PAR1 as a potential tumor-promoting factor in lung cancer [18]. Correspondingly, PAR1 is shown to be indispensible and sufficient to promote tumor growth in a breast cancer model [17]. In the current manuscript, we however show that PAR1 expression levels are not associated with the overall survival of PDAC patients and that PAR1 silencing in pancreatic cancer cells potentiates tumor growth. The observed increase in tumor growth of orthotopically implanted PAR1 knockdown cells is particularly interesting since we previously described that PAR1 depletion in the stroma in fact limits tumor growth [25]. These findings suggest that PAR1 has an opposing activity in the stroma and tumor cells, and that PAR1 activity in the stroma appears to promote tumor growth. The opposite role of PAR1 in tumor cells compared to stromal cells likely explains the lack of association between bulk tumor PAR1 levels and overall survival in PDAC patients. In addition, it further highlights the complexity of pancreatic cancer and strengthens the notion that specific compartments need to be targeted in PDAC for efficient tumor control.

Our investigation of PAR1 expression in PDAC bulk tumors and in micro-dissected tumor cells together with PDAC cell line gene expression datasets shows that PAR1 expression correlates with EMT related genes. Moreover, downregulation of PAR1 in the tumor compartment results in enhanced epithelial characteristics and lower tumor grade. Although the exact molecular mechanism between PAR1 activation and ZEB1 expression are not yet discovered, the increase in E-cadherin expression in all shPAR1 knockdown cell lines with a simultaneous decrease in ZEB1 and the decreased Vimentin expression in PANC-1 shPAR1 cells indicates that PAR1 plays a role in the initiation and maintenance of mesenchymal differentiation. In addition to its role in maintaining mesenchymal characteristics in tumor cells, PAR1 activation induces migration in PANC-1 shCtrl cells, indicating a further shift into a mesenchymal phenotype. Moreover, we observed differences in macro-metastases scores in the KP model between shPAR1 and shCtrl tumors. Altogether, these findings strongly suggest that PAR1 activation on tumor cells initiates mesenchymal differentiation and increases the metastatic potential. Recent studies have shown that suppression and reversion of EMT stimulates proliferation and that growth at the metastatic site is dependent on mesenchymal to epithelial reversion [15, 34]. Furthermore, it has recently been reported that the epithelial status and E-cadherin expression levels mediate cell proliferation *in vitro* and promotes xenograft growth *in vivo* [35, 36]. The mechanistic link between PAR1 and E-cadherin has been demonstrated in a follow up study where doxycycline was proposed as a novel PAR1 inhibitor and doxycycline treated cells exhibited increased E-cadherin expression and a significantly decreased metastatic potential [37, 38]. Considering these findings we might bring an additional explanation for the increased proliferation in shPAR1 tumors *in vivo*. Decreasing PAR1 activity on tumor cell increases E-cadherin expression, thereby diminishing further differentiation and increasing the proliferative capacity. Furthermore, our observations are in line with work of Krebs *et al.* [39], who show less metastasis and more differentiated tumors in ZEB1 conditional knockdown KPC animals as compared to ZEB1 expressing KPC animals. Despite the notion to consider these changes as *bona fide* EMT, we do not observe full conversion of cells into a mesenchymal phenotype. Recent discussions on EMT also report intermediate phenotypes in different cell types and refer to them as “metastable”, implying that these changes can be pushed further or conversely – reversed [40]. Moreover, several intriguing studies indeed show that stable intermediate cell fates with hybrid epithelial and mesenchymal features exist and play a key role in metastasis [41, 42]. Therefore, we conclude that PAR1-induced changes result in a hybrid epithelial/mesenchymal state.

PAR1 silencing in both Panc02 and KP cells results in increased tumor growth *in vivo*, however metastasis is only significantly reduced by PAR1 silencing in the KP model. This could be explained by the fact that the metastatic potential of grafted wildtype Panc02 cells is low, not allowing a further decrease to become evident. Indeed, Panc02 cells have been suggested to have a limited metastatic phenotype [43]. Finally, the different genetic background of the used tumor cell lines may contribute. KP cells are both *Kras* and *Tp53* mutant whereas Panc02 cells are *Kras* wildtype [43, 44]. Considering the importance of *Kras* in human pancreatic cancer [45] we focused on the KP model for detailed experimental characterization of PAR1 mediated cell state transitions.

As opposed to the growth inhibitory effect of PAR-1 *in vivo*, PAR-1 deficiency does not seem to affect proliferation *in vitro*. Obviously, 2-D cultures do not accurately mimic the complex nature of stroma-rich pancreatic tumors and indeed stromal components play crucial roles in cancer cell proliferation. In addition, the *in vitro* experiments are performed in growth factor-rich fully oxygenated conditions that may obscure the effect of PAR1 on proliferation under growth factor and/or oxygen depleted circumstances that exist *in vivo*. Finally, the growth advantage of PAR1 deficient cells *in vivo* is observed after 4 weeks and it may well be that small differences in growth rate are not observed on a short time scale *in vitro*. Overall, this underscores that conclusions based on *in vitro* proliferation experiments may not accurately reflect *in vivo* results and should be interpreted with care.

Several clinical studies have evaluated the potential clinical efficacy of anticoagulants in pancreatic cancer patients. Indeed, in a retrospective analysis of patients who received chemotherapy for advanced pancreatic adenocarcinoma the addition of low molecular weight heparin (LMWH) to standard chemotherapy significantly improved survival in patients with locally advanced or metastatic pancreatic carcinoma [46]. Opposed to these studies suggesting anticoagulants may increase overall survival of pancreatic cancer patients, a large randomized-placebo controlled trial did not show any benefit of LMWH in pancreatic cancer patients [47]. Our data showing that tumor cell PAR1 limits pancreatic cancer progression may provide an explanation for the disappointing efficacy of anticoagulants in PDAC. Indeed, thrombin is the prototypical PAR1 agonist and thrombin inhibition will thus inhibit PAR1 signaling on tumor cells, suppress mesenchymal transition, and enhance tumor cell proliferation. In line with this notion, we previously showed that thrombin inhibition is less effective in the setting of pancreatic cancer as compared to stromal PAR-1 depletion and we hypothesized this may be due to the counteracting effect of thrombin-PAR1 signaling on tumor cells [48].

Overall, we show that, against its anticipated oncogenic role, tumor cell PAR1 limits PDAC progression by enhancing a mesenchymal phenotype of pancreatic cancer cells. This implies that PAR1 plays a dual role in pancreatic cancer progression and that any therapeutic strategy focusing on PAR1 should be on the stromal compartment. Such compartmentalized PAR1 targeting might be challenging although PAR1-dependent biased signaling, in which different agonists induce different functional responses, may provide an opportunity. Indeed, identifying and targeting PAR1 agonists that drive tumor progression in the tumor compartment, without affecting tumor inhibitory PAR1 signaling on tumor cells, would be a promising strategy to pursue.

## MATERIALS AND METHODS

### Animals

C57BL/6 mice (Charles River Laboratories) were housed at the animal facility of the Academic Medical Center of Amsterdam. All mice had access to food and water ad libitum. Institutional Animal Care and Use Committee of Academic Medical Center approved all animal experiments according to protocol number DIX102373 and DIX107AA.

### Orthotopic pancreatic cancer model

Cultures of Panc02 (kindly provided by Dr. Schmitz, Universitatsklinikum Bonn, Bonn, Germany) and KP cells (derived from pancreatic adenocarcinomas from p48-CRE/LSL-KRAS/P53^flox/flox^ KPC mice, kindly provided by Dr. DeNardo, Washington University Medical School, St. Louis, MO) were trypsinized at 80% confluency, pelleted, washed twice in phosphate buffered saline (PBS) and re-suspended in 0.9% sterile saline (Sigma, St Louis, MO). During tumor inoculation mice were given with Tamgesic (0.05 mg/kg) and anaesthetized with isoflurane (2% in CO_2_). Tumor cells (4 × 10^5^ cells per animal) were injected directly into the tail of the pancreas of 8- to 10-week-old mice essentially described as before [25]. Mice were evaluated for changes in body weight and signs of discomfort or morbidity, and they were euthanized 4 weeks after tumor cell injection. Whole pancreata were removed and weighed, followed by fixation in 4% formalin and embedding in paraffin for further analysis.

### Cell culturing

Murine KP and Panc02 cells and human PANC-1, Capan-2, and MIA PaCa-2 cells (ATCC, Manassas, VA) were cultured in high glucose (4.5g/mL) DMEM, 10% fetal bovine serum (FBS), L-glutamine (2mM), penicillin (100 units/mL), and streptomycin (500 μg/mL) (Lonza, Basel, Switzerland) according to routine cell culture procedures. Cells were incubated in 5% CO_2_ incubators at 37° C. Human cell lines were authenticated by STR profiling (Promega PowerPlex) and tested for mycoplasma by PCR monthly.

### Lentiviral silencing of PAR1

PAR-1 knock down cells were established as described before [25]. Briefly, PAR-1 (clone TRCN0000026806 for murine cells and clone TRCN0000003690 for human cells) and control (clone SHC004) shRNA in the pLKO.1-puro backbone were purchased from the MISSION shRNA library (Sigma-Aldrich, St. Louis, MO). Lentivirus was produced by transfecting HEK293T cells with 3rd generation transfer and packaging plasmids pVSV, pMDL, and pRES using Lipofectamine 2000 (ThermoFisher Scientific, Waltham, MA). 48 and 72 hours after transfection, supernatant was harvested and 0.45 μm filtered (Millipore, Billerica, MA, USA). 75% confluent PANC-1, MIA PaCa-2 and Capan-2 cells were transduced with 20 μl lentivirus and incubated for 24 h. Transduced cells were selected with 2 μg/ml puromycin (Sigma, St.Louis, MO) for 72 h.

### PAR1 overexpression

PANC-1 cells were transfected using Lipofectamine 2000 according routine procedures (ThermoFisher Scientific, Waltham, MA) with *pcDNA3.1(+)* plasmid containing *PAR1-P2A-eGFP* (Genscript, Piscataway, NJ) and *pcDNA3.1(+)* coding for *eGFP* as control (Addgene, Cambridge, MA). 48 hours after transfection, GFP-positive and GFP-negative single cells were sorted using a Sony Cell Sorter SH800S (Sony Biotechnology, San Jose, CA). Cells were sorted directly into RNA lysis buffer of the RNeasy Mini Isolation Kit (Qiagen, Hilden, Germany) after which RNA was isolated following manufacturer's instructions.

### Flow cytometry

Cells were harvested with 5 mM EDTA and washed with FACS buffer (1% FBS/PBS). Cells were stained either with PAR1 (ATAP-2: sc-13503, Santa Cruz Biotechnology, Dallas, TX) or E-cadherin (24E10; Cell Signaling Technology, Danvers, MA) as primary antibodies and anti-mouse APC (550826; BD, Franklin Lakes, NJ) and anti-rabbit Alexa 488 (A-11008; Invitrogen, Carlsbad, CA) secondary antibodies with 1:400 dilution for each antibody. For PAR1 inhibition on PANC-1, MIA PaCa-2 and Capan-2 wildtype cells, 250 nM Vorapaxar (SCH530348, Adooq Biosciences, Irvine, CA) was added and cells were analyzed 48 hours later. In all assays, samples were prepared following the manufacturer's instructions, and analyzed on FACS Canto II (BD, Franklin Lakes, NJ). Data were analyzed using FLOWJO v10 (FlowJo LLC, Ashland, OR). Cells were gates initially based on FCS and SSC for the main cell population and later FCS-H vs FCS-W for single cells. APC or FITC positive populations were gated on single cell population based on antibody control samples.

### Quantitative real-time PCR

Total RNA was isolated with TriReagent (Sigma, St. Louis, MO) and chloroform separation with repeated ethanol washes. cDNA was synthesized from DNase treated total RNA by using M-MLV-RT enzyme (Promega, Leiden, Netherlands) with random hexamers (Qiagen, Hilden, Germany). Real-time quantitative RT-PCR was performed with Sensifast SYBR No-Rox Kit (Bioline, London, UK) on a lightcycler LC 480 II (Roche, Basel, Switzerland). Relative expression of genes was calculated using the comparative threshold cycle (dCt method) and were normalized for expression of reference gene TBP. Primer sequences of the analyzed genes are; hTBP (fw 5′-ATCCCAAGCGGTTTGCTGC-3′; rv 5′-ACTGTTCTTCACTCTTGGCTC-3′), hF2R(PAR1) (fw 5′-GCAGGCCAGAATCAAAAGCAACAAATGC-3′; rv 5′-TCCTCATCCTCCCAAAATGGTTCA-3′), hCDH1 (fw 5′-TGGAGGAATTCTTGCTTTGC-3′; rv 5′-CGC TCTCCTCCGAAGAAAC-3′), hZEB1 (fw 5′-GCAC AAGAAGAGCCACAAGTA-3′; rv 5′-GCAAGACAAGT TCAAGGGTTC-3′), hVIM1 (fw 5′- AGTCCACTG AGTACCGGAGAC-3′; rv 5′- CATTTCACGCATCTG GCGTTC-3′).

### Western blot

PANC-1 cells were seeded in 6-well plates in DMEM supplemented with 10% FCS. After 48 hours, cells were lysed in RIPA buffer and Western blots were performed as described before [49]. In brief, protein samples were boiled in Laemmli buffer with 3% beta-mercaptoethanol for 10 minutes at 95° C, separated by 10% SDS-PAGE and transferred to a PVDF membrane (Millipore, Billerica, MA). Membranes were blocked for 1 hour in 4% milk in TBS-T and incubated overnight with antibodies against a-tubulin (1:1000, Santa Cruz, CA) or E-cadherin (1:1000, 24E10; Cell Signaling Technology, Danvers, MA) at 4° C. All secondary antibodies were horseradish peroxidase (HRP)-conjugated from Dako Cytomation (Glostrup, Denmark) and diluted according to the manufacturer's instructions. Blots were imaged using Lumilight Plus ECL substrate from Roche (Almere, The Netherlands) on a LAS 4000 imager from Fuji (FujiFilm, Tokyo, Japan).

### Kaplan–Meier survival analysis

The following datasets were used: The Cancer Genome Atlas (TCGA)-PDAC [50], GSE17891 [51], GSE62452 [52], GSE15471 [53], GSE21501 [54]. Kaplan–Meier survival analysis was based on median PAR1 (*F2R*) expression. Kaplan–Meier analysis and gene expression data were collected and processed for use in the AMC in-house R2: Genomics Analysis and Visualization Platform (http://r2.amc.nl). For visualization of gene expression, data were plotted in GraphPad Prism 7.0 (GraphPad Software Inc, La Jolla, CA, USA).

### Gene set enrichment analysis

Datasets used were the tumor expression datasets GSE28735 [55], GSE16515 [56], The Cancer Genome Atlas (TCGA)-PDAC [50], GSE62452 [52], GSE21501 [54], micro dissected tissue expression data: E-MEXP-1121 [57] and cell line expression data: GSE36133 [58]. GSEA software (Broad Institute, Cambridge, MA, USA) was downloaded from the Broad Institute website (http://www.broad.mit.edu/gsea/) and signature sets for cancer mesenchymal transition [59], and hallmark epithelial to mesenchymal transition (Broad Institute) were downloaded from the Molecular Signature Database (MSigDB). Expression datasets were compiled with annotated gene names (.gct), samples were segmented for median PAR1/F*2R* expression (i.e. high and low) as phenotype label files (.cls), and signature sets were assembled (.gmx). One thousand permutations were run on the phenotype. Datasets were not collapsed to gene symbols (collapse to gene symbols = false) in the GSEA software.

### Immunohistochemistry

Histological examination was performed essentially as described before [25]. Briefly, the excised tumor was fixed in formalin, embedded in paraffin and 4-μm-thick slides were subsequently deparaffinized, rehydrated and washed in deionized water. Slides were stained with hematoxylin and eosin (H&E) according to routine procedures. For immunohistochemistry, endogenous peroxidase activity was quenched with 0.3% hydrogen peroxide for 15 min at room temperature, with antigen retrieval for 10 min at 100° C in 10 mM sodium citrate buffer, pH 7.4. Slides were blocked for 10 min with 5% normal goat serum. Primary antibodies against, Anti-alpha smooth muscle Actin antibody (ab5694; Abcam, Cambridge, UK), E-cadherin (24E10; Cell Signaling Technology, Danvers, MA), or Ki67 (1:500, clone Sp6; Neomarkers, Fremont, CA), were added for overnight incubation at 4° C. Slides were subsequently incubated with appropriate HRP-conjugated secondary antibodies and DAB staining was used to visualize peroxidase activity. Slides were photographed with a microscope equipped with a digital camera (Leica CTR500, Leica Microsystems, Wetzlar, Germany). The number of Ki67 positive cells were counted in five different fields at 20× magnification, counting was performed with ImageJ and the expressed count per image.

### Immunofluorescence

Cells grown on coverslips were fixed with 4% formaldehyde. F-actin was stained with (1:1000) Acti-stain 535 (rhodamine) Phalloidin (Tebu Bio, Heerhugowaard, Netherlands), (1:400) ZEB1 antibody (HPA027524, Atlas Antibodies, Sigma, St. Louis, MO), (1:400) E-cadherin antibody (24E10; Cell Signaling Technology, Danvers, MA), (1:1000) DAPI (ThermoFisher Scientific, Waltham, MA), 1:400 secondary antibody Alexa488 conjugated anti-rabbit IgG (ThermoFisher Scientific, Waltham, MA). All reagents/antibodies were dissolved in 1% bovine serum albumin (Sigma, St. Louis, MO) in PBS with 0.1% Triton X-100 (Sigma, St. Louis, MO) in PBS. Images were acquired on a Leica SP-8 Confocal Microscope (Leica, Wetzlar, Germany) at 63X magnification. LUT values of channels were improved for better visualization in LAS AF software (Leica, Wetzlar, Germany).

### Calcium-flux assay

Calcium signaling responses were analyzed using the Fluo-4 Direct™ Calcium Assay Kit (Invitrogen, Carlsbad, CA) as described before [27]. Cells were challenged with thrombin (1 U/ml) or PBS. Ca2+ flux was monitored for the indicated time points on a Bio-Tek HT Multi-Detection Microplate Reader (Winooski, United States).

### MTT cell proliferation assay

Cells at 70% confluency in 96-well plates were serum starved overnight after which cell viability was determined using a 3-(4,5-dimethylthiazol- 2-yl)-2,5-diphenyltetrazolium (MTT) assay at 0, 24, 48 and 72 hours according to routine procedures. Measurements were performed on a Synergy HT Biotek Microplate Reader (Biotek Instrumens, Winooski, VT) at 560nm. Fold changes were calculated based on optical density at *t* = 0.

### Wound-scratch assay

Cells were seeded onto six-well plates and maintained in 10% FCS/DMEM until confluence. Next, cells were serum staved overnight and a scratch was created in the center (on the vertical axis) with a p200 pipette tip. Cells were incubated up to 72 h with serum-free DMEM with 25 μM PAR1 agonist peptide TFLLR-NH_2_ (GL Biochem, Shanghai, China) or solvent control (PBS) as mock. Scratched area were scanned every 24 hours at 4X magnification with EVOS^®^ FL Cell Imaging System. Wound area analysis was performed at fixed locations (400 × 400) along the scratch area at each time point. Wound area at t = 0 is taken as 100% and the changes in wound area at each time point was calculated based on the difference from the area at t = 0. Three independent replicates were included for each measurement (*n* = 3).

### Statistical analysis

Data were presented as Mean ± SEM. Statistical analysis was performed with built-in analysis tool of GraphPad PRISM 7.0. For further details see figure legends.

## SUPPLEMENTARY MATERIALS AND FIGURES


