# Supplementary material for: PAR1 signaling on tumor cells limits tumor growth by maintaining a mesenchymal phenotype in pancreatic cancer
Source: Oncotarget. 2018 Aug 10;9(62):32010–23. doi: 10.18632/oncotarget.25880 (PMC6112838; doi:10.18632/oncotarget.25880)
Supplement: Supplementary file 1 [file oncotarget-09-32010-s001.pdf]

PAR1 signaling on tumor cells limits tumor growth by maintaining a mesenchymal phenotype in pancreatic cancer

SUPPLEMENTARY MATERIALS

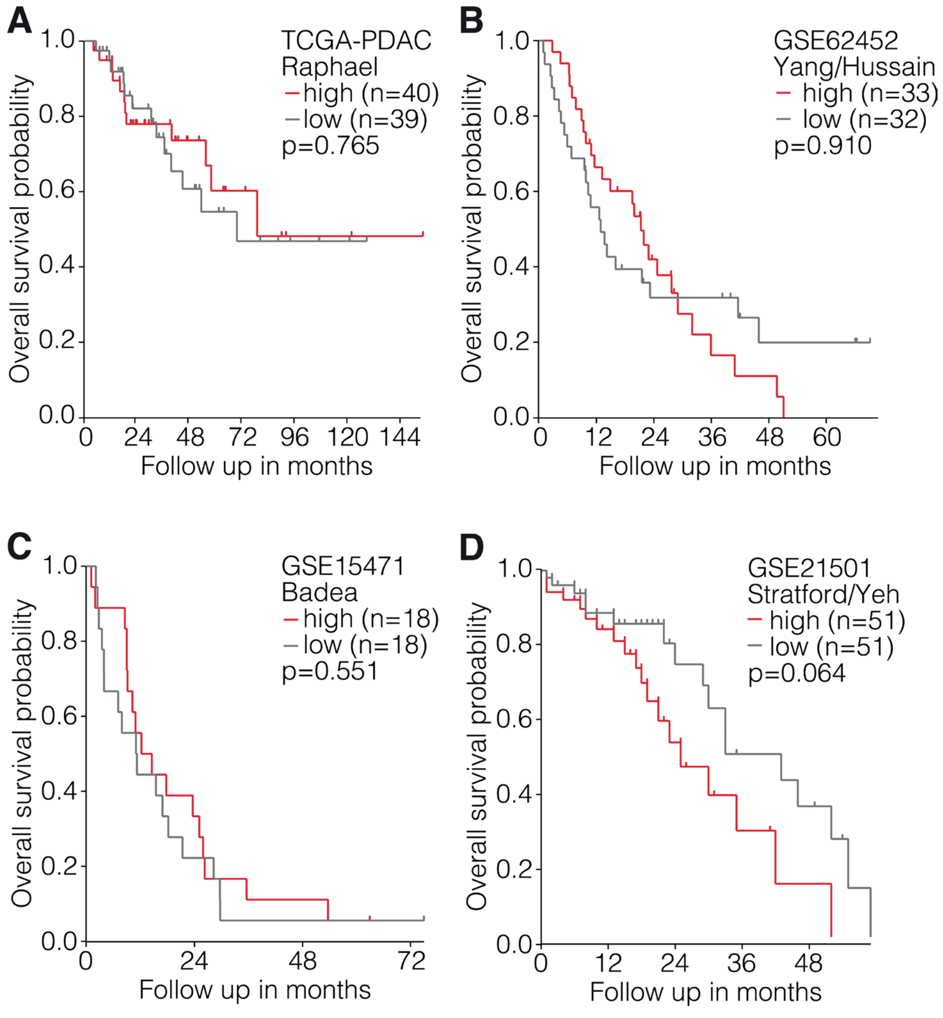

Supplementary Figure 1: Kaplan-Meier survival analysis of PDAC patient sets based on PAR1 expression.

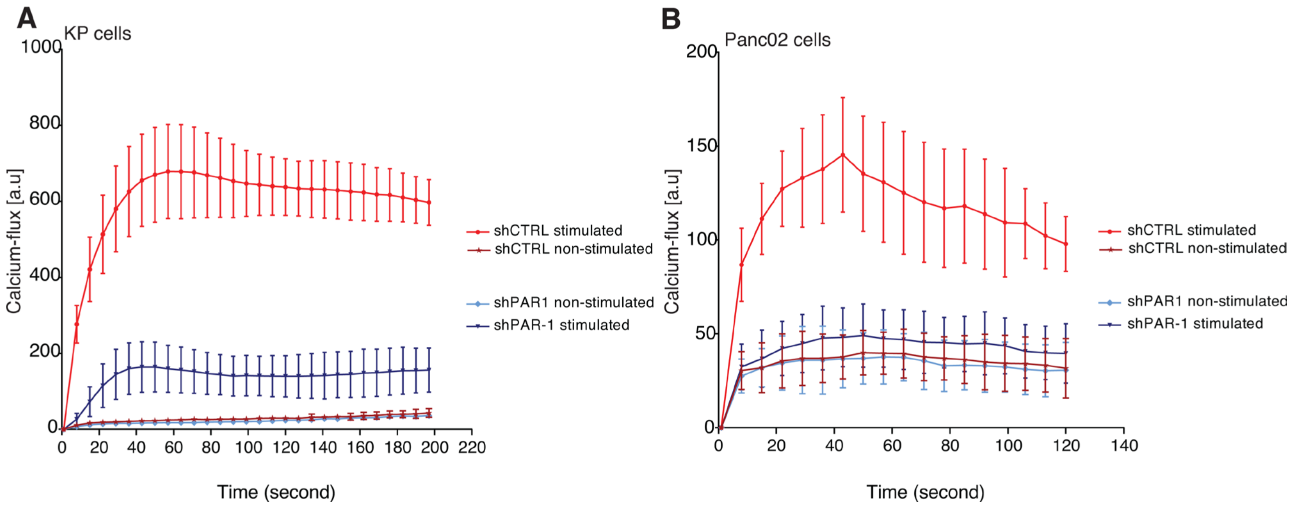

Supplementary Figure 2: Functional confirmation of short hairpin mediated PAR1 knockdown of murine cell lines with calcium flux assay.

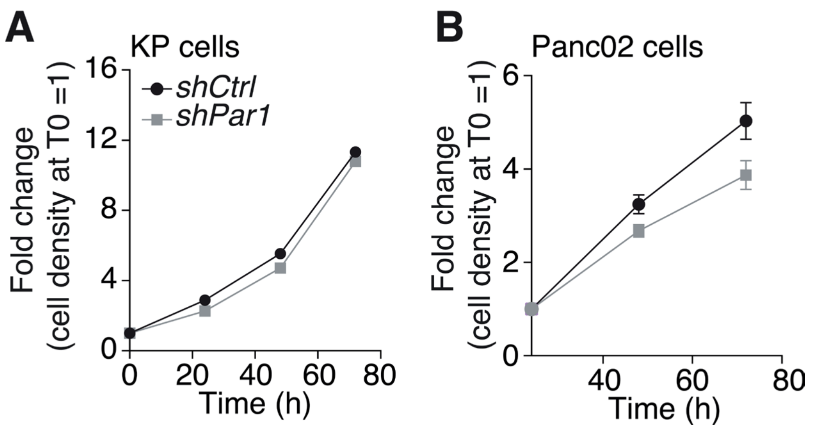

Supplementary Figure 3: Proliferation rate comparison of murine shPAR1 and shCtrl cell lines.

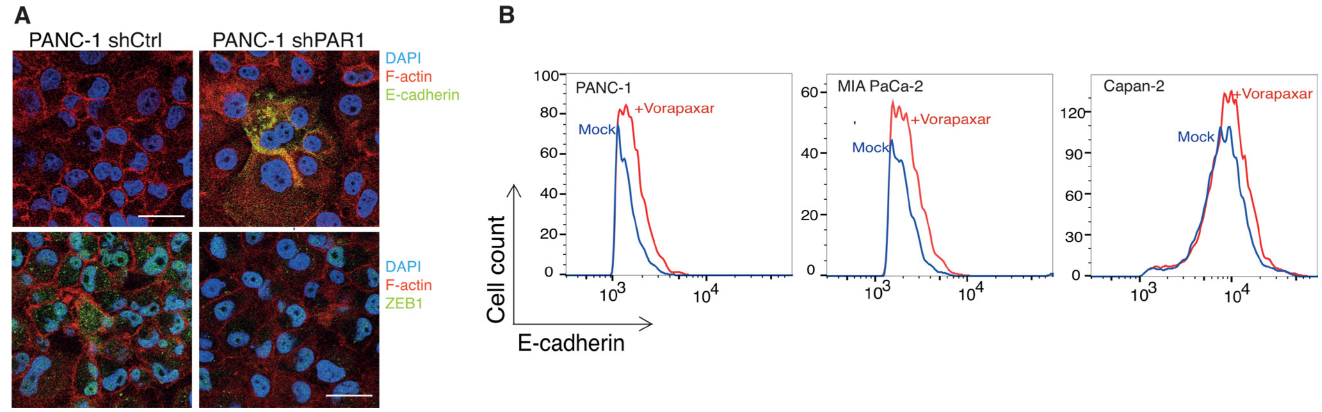

Supplementary Figure 4: PAR1 activity and E-cadherin expression.

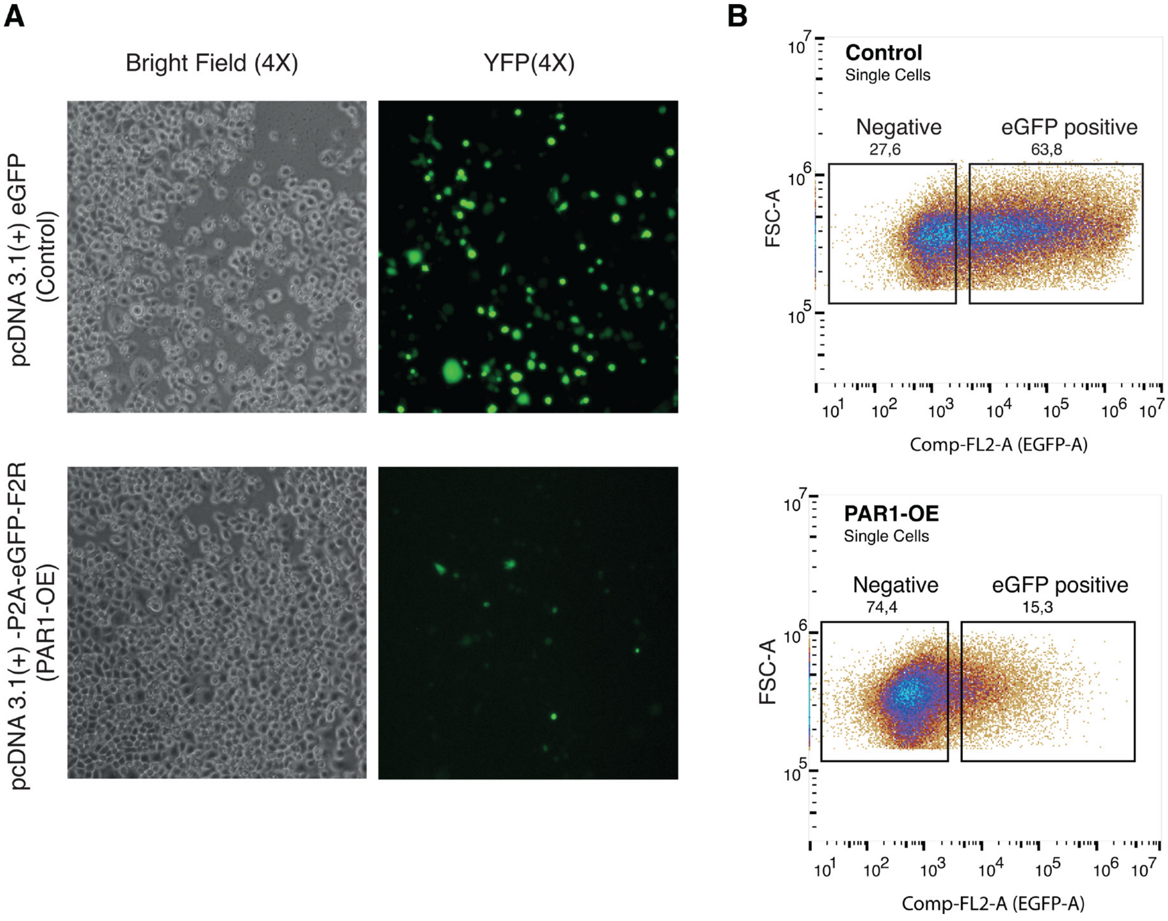

Supplementary Figure 5: Selection and sorting of PAR1 overexpressing cells.
